# Understanding the Significance of Listening to Older People's Life Stories in Whole Person Care—An Interview Study of Nurses in Gerontology

**DOI:** 10.1177/23779608231164077

**Published:** 2023-03-17

**Authors:** Kristin Ferstad, Linda Rykkje

**Affiliations:** 1 VID Specialized University, Faculty of Health Studies, Bergen, Norway

**Keywords:** Martinsen’s caring theory, hermeneutics, interview, thematic analysis, gerontological nursing, life story narrative

## Abstract

**Aim:** The purpose of this study was to explore the significance of performing a life story interview for gerontological nursing students. **Method:** The study had a qualitative exploratory design, focusing on hermeneutical understanding using thematic analysis. Seven nurses in older people nursing were interviewed. **Findings:** Two main themes emerged from the analysis: “Engaging fellowship” and “Understanding the importance of life stories.” The participants experienced increased engagement and fellowship with their patients after the life story interview; the change in their perspective was characterized by renewed interest, connection, and recognition of the individual person. The participants also gained a deeper understanding of the significance of listening to an older person's life story narrative, and this was expressed through them gaining an understanding of people's actions, achieving an altered mindset, gaining a greater generational understanding, and integrating a life story focus in their everyday professional life. **Conclusion:** Knowledge of human life and stories makes older people's situations easier to understand; this insight affects how we as nurses think about others. Seeing each patient as an individual and unique person and being aware of this in daily care is essential for nursing.

## Introduction

Services for older people in Norway include institutional and home health care services; these are provided by municipals and are based on caring needs and are part of the welfare system. Nurses in municipal healthcare service report experiencing increased responsibilities, new tasks, and workload, thus having less time to provide good quality care (Bratt & Gautun, 2018; Gautun, Øien & Bratt, 2016). They have constant time pressure; their responsibilities increase simultaneously as the available time for each patient is reduced (Berge & Eilertsen, 2020). This situation increases the risk of engaging in task-focused work and decreases compassionate care (Sharp et al., 2018). The purpose of the Norwegian quality reform *Leve hele livet* [*Living your whole life]* (2017–2018) is to provide good quality care for older people (Meld, 2017–2018); that quality of care entails that caregivers not only see their patients’ illnesses and disabilities but also rather provide for them as *whole* persons.

### Literature Review

To enable whole person care, the use of life story work in older people care has received increased attention in recent years. In the Norwegian Government's Dementia Plan (2020), *A more dementia-friendly society*, the “Life story template” is highlighted as a tool for good quality care. The template provides a short text with an overview of the person's life experiences and preferences (Hopen, 2015). A similar tool for life story work is the British “This is me” leaflet. These methods have also been criticized. McKeown, Ryan, Ingleton and Clarke (2015) ask: “Who owns the stories? Who decides what should be included, excluded and focused on?” Thus, there are reasons to question what happens to the individual's unique story when it is fragmented and systematized in this way (Simonhjell & Hellstrand, 2019). However, the life story interviews performed by the participants in this study were done with open questions and not according to a template, which provided the older persons an opportunity to narrate freely about their life experiences.

The most common method of studying a culture is listening to the stories of ordinary people (De Chesney, 2015). How we understand and present ourselves and the world around us can never be separated from our larger cultural and social context (Medeiros, 2014). This study focuses upon nurse's life story work and how it might benefit older people and improve care provision. Life story work can contribute to “care for the whole person” by providing a way to gain insight into what is important and valuable for the individual person (Fossland & Thorsen, 2010; Medeiros, 2014). A life story narrative provides nurses with an opportunity to look through the eyes of the other person, and, in this way, nurses share the patient's experiences. Blix et al. (2021) put forward the claim that care itself can be understood as a narrative endeavor, where the nurse is an active partner engaged in dialogue with the older person while being open to narrative expressions in everyday life and actively engaging in storytelling activities. Sharing stories can also enable the older person to develop his/her own perspective upon his/her life. The strength of the narrative lies in its ability to engage nurses, as proposed by Hovland (2011, p. 17): “*Stories do not influence through arguments, but by showing possibilities and identification with the characters in the story*.” Hovland (2011) further argues that it is crucial for the significance of the story that it resonates with the listener and is recognizable in relation to other people's life experiences.

### Theoretical Perspective

This study is based on the Norwegian nursing philosopher Kari Martinsen's caring theory. In Martinsen’s thinking, human beings are born as dependent and relational individuals, and thus care is a fundamental part of human life. Caregiving in nursing represents the encounter with another vulnerable human being and is learned through engaging in practical experience and receiving guidance in concrete situations (Alvsvåg, 2021). On this account, the body is understood as a whole entity with spirit and mind; therefore, when nurses are interacting with a patient, “sensing” cannot be avoided (Alvsvåg, 2021). Martinsen portrays how *sensuousness* makes the nurse able to listen and be responsive to the signals of the patient while attentively engaging with them. *Sensuousness* requires awareness and willingness—the nurse must be engaged with and interested in the other person (Martinsen, 2000). *Sensuousness* also helps the nurse to *hold back*. Martinson's concept of *holding back* is an important contrast to the interpretation of nurses as “experts” or knowing how to quickly handle the situation and what to do or say. In *holding back*, the nurse listens and is open to the given situation, having the courage to be present, quiet, and stay in uncertainty (Martinsen, 2003). This means that caring cannot be done too immediately by doing or saying something. The nurse is *holding back* so that the patient can appear clearly, and the patient's person and perspective become the center of the situation (Martinsen, 2010).

### Purpose

The purpose of the study was to explore how nurses enrolled in further education in gerontology experienced the significance of conducting a life story interview with an older person.

## Methods

### Design

The study had a qualitative, exploratory design (Malterud, 2017), focusing upon a hermeneutic understanding.

### Research Question

The following research question was examined in the study:

What is the significance of a life history interview for nurses in older people care?

### Sample

Data were collected through individual conversations with the participating nurses. Qualitative research interviews are appropriate for understanding the world from the perspective of the interviewees (Kvale & Brinkmann, 2017). The participants in the study were recruited from a further education program in gerontology. The study was presented to two different classes (about 40 students were informed of the study), and the participants took the initiative to contact the interviewer.

### Analysis and Interpretation

The data material was analyzed based on Braun and Clarke's (2006) thematic analysis and was inspired by Gadamer's (2004) hermeneutic philosophy. Gadamer's (2003, 2004) philosophical hermeneutics was furthermore used in the search to gain an understanding of the importance of listening to older persons’ life stories. In hermeneutic thinking, the world around us is interpreted.

We interpret our experiences in life based on our preconceptions, which for Gadamer is a necessary ground that enables understanding. To understand, we must have already understood something else. This is what Gadamer calls “the structure of understanding” (Gadamer, 2003, p. 26). If we were without preconceptions, we would be unable to process impressions. Gadamer's (2003) understanding is not only intellectual but also touches body, feeling, and experience. The preunderstanding in this study is based on the authors’ experience in older people nursing and is also placed within the framework of Kari Martinsen's nursing theory.

## Results

### Sample Characteristics

Seven female nurses aged 26–55 years (average: 37 years) agreed to participate. Their experience working as nurse was between 3 and 24 years (average: 7 years). All participants worked in older people care (nursing homes and home health nursing) and they completed the life story interview in the first semester of their further education.

### Research Question Results

**Figure 1.**
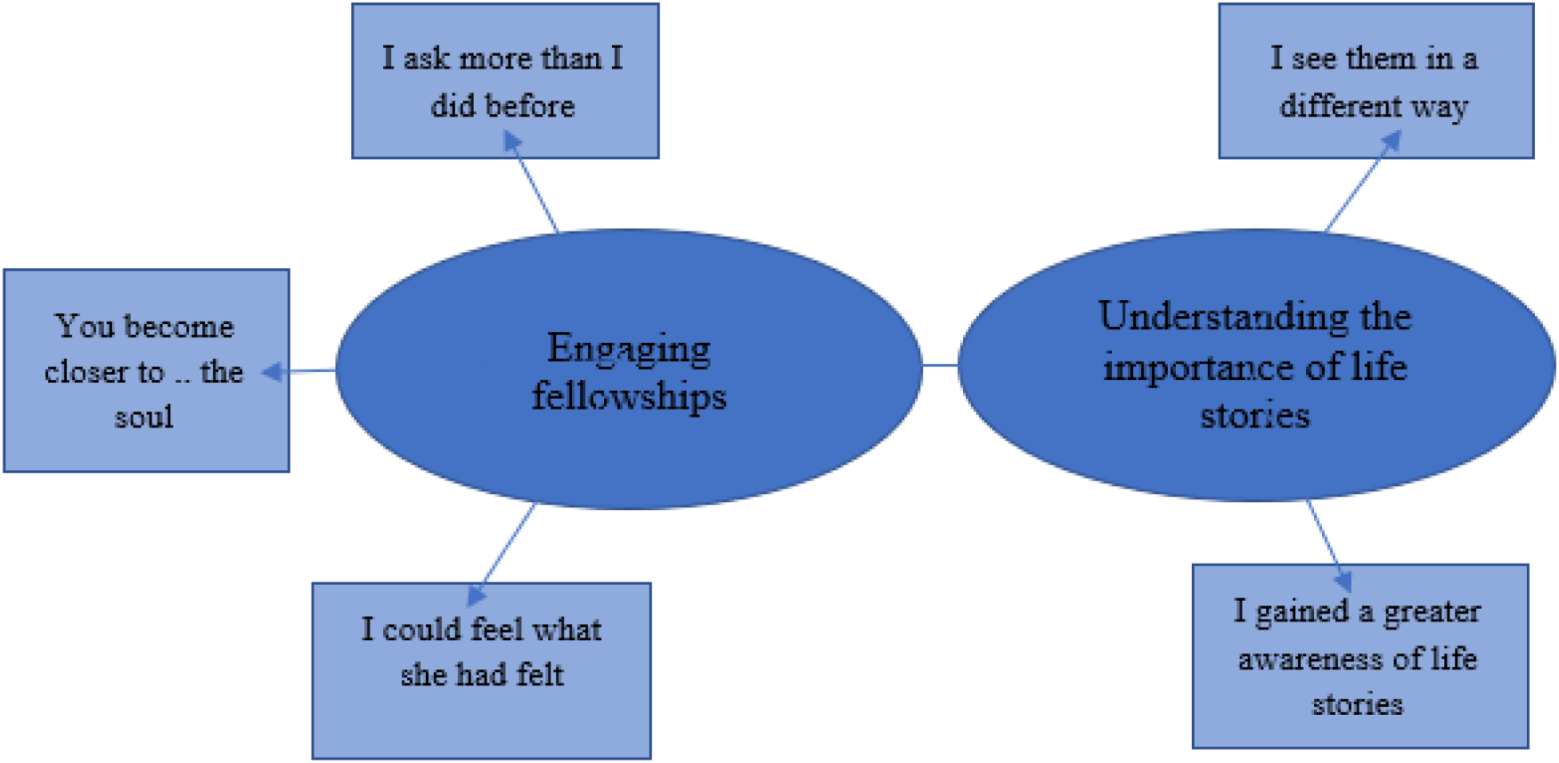
Themes and subthemes.

The study's results are presented in two main findings: “Engaging fellowships” and “Understanding the importance of life stories,” with five subthemes (see Figure 1).

### Engaging Fellowship

The Engaging fellowship theme has three subthemes that relate to the life story interview and the person that the participants interviewed; the nurses’ focus was on the affect that listening to the story had on their everyday work. Our understanding is that the life story fostered engagement and experiences of fellowship between the nurse and the older person; this insight was repeated in all the interviews with nurses. One of the participants, Ingrid, expressed how the story broadened her perspective:

I was speechless when she told her story and I was allowed to listen…it was a whole story that I knew nothing about…from being this older lady who had a broken hip and cancer…and sort of…that I knew, I only knew those things…to also become a whole story…a whole person…much more than the frailty that I knew about, right?

#### I Ask More Than I Did Before

All the participants pointed out that they ask more questions of and listen more to patients in the work context than they did before the life story interview. Some also explicitly note that this is based on renewed interest in their patients. One of the participants, Guri, put it this way: “*I suddenly became curious about those [patients] I work with…I started to think: who are you?”*

Where they used to ask questions more on “*autopilot,*” they now described having more genuine interest in their patients. The nature of the conversations they had with their patients was influenced by this renewed interest. An example is that the conversations they had with their patients related less to nursing or diseases. They now focused more on the patient's previous life and what was important in both the past and present of the individual. Ingrid described a sort of freedom in being “*allowed*” to be a person who meets another person, and she expressed that having that feeling reduced the pressure she had felt of always being in the role of a nurse. Another participant, Natalia, emphasized that she meets patients with a more “*open mind*” and was less “*stuck*” in the nursing role.

The participants also emphasized the importance of knowing what questions to ask and how to facilitate a good conversation. Guri described how she often asked her patients, “*how are you today?”* and would receive unengaging answers. However, after the life story interview, she asked different questions and thus the conversation has become more interesting for both parties. She pointed out: “*you know…they have a whole life to tell about.”* Some of the participants also pointed out that they have more issues to talk about, which contributes to them having more engaging conversations with their patients.

Prioritization of time was a constant concern among the participants. Lene, who works in home healthcare, told that she changed her ways after the interview: “*I probably prioritize time a little differently when I am at the patient's home…I spend more time on the person instead of the patient.”* Furthermore, the participants found that conversing, even under time pressure, can be successful if you know what to ask about to facilitate a good conversation. Although the participants often expressed that they do not have enough time to thoroughly speak with their patients, they could still pick up the thread of the conversation the next time they visited the patient. This is pointed out in Hanne's remark: “*I discovered that…you don’t really need that many minutes to make contact.”*

#### You Become Closer to the Soul

Furthermore, good conversations contributed to good relations. Mari described the experience of listening to a life story as coming closer to the “*soul,”* before quickly commenting that this was a “*silly word to use.”* The word is still descriptive, and several other participants also found it difficult to put their changes into words. They described a sense of deeper contact, increased engagement, and experience of fellowship with patients. A patient's individual personality and identity became visible through the life story, as expressed by Ingrid:

I think, there is a huge difference between a COPD-patient and a sailor who has travelled across the seas and experienced…done so much and shown the tattoo and the sailor's coffin and told about his life…they get an identity. Working with a person versus working with a diagnosis…it`s completely different.

Others point out that health services are arranged in a way that treats people as a chore. For example, Thea emphasizes the importance of being present as a human being: “*I think we become more human ourselves by seeing humans, not diagnoses or work tasks.”* A meeting between two fellow people makes the patient relationship less one-sided. The fact that the relationship goes both ways also helps build trust, as Guri explained: *“I see that, when I give a little of myself, and they give of themselves—they become so much more confident in me and they open up and dare to ask and tell*.” The participants also pointed out that the relationship becomes more exciting and engaging when it is more equal. The participants now share more from their own lives, and they described this as having been caused by a renewed interest in and ability to be present with the older person. Lene also described being more open for learning in relation to patients: “*They can tell me so much and they have so much wisdom and insight…life experience…life story…I can learn so much from them…I see that now.”*

#### I Could Feel What She Had Felt

Several of the participants described that they became more emotionally engaged following the life story interview, not only with the person they interviewed but also with other patients who later told stories from their lives. The participants expressed that the story they listened to was “*vivid*” and recognizable. Mari described this with the theme “*I could feel what she had felt.”* Another example was Hanne, who described this feeling by stating: “she came under my skin in a fantastic way…when I read the interview, I feel like I can read some of her thoughts.”

The nurses experienced an increased ability to immerse themselves in patients’ situations and mindsets and they recognized themselves or someone they loved in their patients’ life stories. The participants reflected on how life stories made it easier to see things from the patient’s point of view. Guri spoke about this by saying: “It gives you a kind of context…which makes it easier to understand…their situation.” Also, Ingrid described the insights that the life story interview gave her, and she explained that this history gives her a framework for handling future situations: “when you know the story, it is somehow much easier to understand what happens…in the present.” Some respondents also highlighted that you have a better basis for medical practice as a nurse when you can understand the patient´s situation based on their life story. This was expressed by Lene in the following:

You see the person much better…clearer…for example, anxiety: What lays beneath that really, then you can almost hear the life story and maybe you can find some of the core of the anxiety problem, right? It's easier to see clearly somehow.

### Understanding the Importance of Life Stories

This theme has two subthemes. The theme “I see them in a different light” describes the participants’ direct experiences with the life story interview as well as what the participants described as the consequences of the interview experience for their everyday work. “I became more aware of the life story” is a theme directly linked to the life story interview and the person the participants interviewed. The main thrust of this theme is that hearing a life story promotes understanding; this is true for the person sharing their story and for life story work in older people care. This understanding is an extension of the interest in and experience of fellowship and engagement we noted in our discussion of the previous main theme. This is illustrated by Hanne:

You might get a completely different relationship with them [patients], and I think that the more I know them, the more I sort of get involved to find out more about them, the more I will be able to understand them, why they react like they do…and why they act like they do…the story tells me a lot.

### I See Them in a Different Way

The participants refer to the experience of having a more holistic understanding of the patient, and they described this as a way of zooming out to have a greater perspective when meeting patients. This was reflected in the following statement by Lene:

The way I meet older patients…I see them in a different way. I think that…this is a person with an incredibly long life behind them and they have been shaped by all the things that have happened through their life, small stuff and big stuff.

Several of the participants described having a renewed understanding of the older generation after participating in the interviews. Life story work led to a realization that the older person's life circumstances and upbringing were very different from their own. Factors such as war, technological change, living conditions, access to education, and lack of equality were mentioned. One of the youngest participants, Thea, said that gaining an understanding of the differences in growing up back in the old days made it easier to understand the differences in the thoughts and attitudes of older people in the present. Some nurses also demonstrated awareness about how society's stigmatizing descriptions of old people affect their own attitudes. This was reflected in Gurís response:

One becomes influenced by the media, right? There is many such…the Age Wave and…a lot such negativity related to it…also one forgets that…they are just as different as everyone else…[The life story interview] has probably made me more like…expanded my perspective in working with older people.

The participants also described having a better understanding of the individual patient's point of view in everyday settings and found it important to respect “little things” that are valuable to the individual patient. Examples given were when patients collected specific items, patients having very specific routines, or patients being very careful about eating all their food. This was expressed in the following way by Lene: “*Such small things they do…maybe…small things that make them angry…or small things they appreciate very much…you give it more weight in a way…because you think in slightly different ways.”*

#### I Gained a Greater Awareness of Life Stories

Several of the participants expressed that they became more aware of the value of the older person when that person was able to share their story. The nurses reflected on how important it was to be seen and listened to and described how it seemed to be good for the narrator to “*speak out*” about their experiences. The participants expressed gratitude for being allowed to listen to the life story. It was pointed out that the narrator in the life story interview was free to tell and omit parts, as they were invited to provide only the information they wanted. It was the narrator who was in control, and their story was not influenced by the patient's relatives or the nurse's interpretation, which may otherwise be the case. One participant (Mari) expressed:

[The life story interview] provides a sort of special insight into how different events and experiences have affected values…attitudes…reactions they have and…what is important to them…One gains a…deeper understanding of a human beings’ life and what is individual and unique for that individual.

The nurses were surprised that the life story interview affected them as much as it did. They emphasize that seeing the person behind the patient was not new to them but that this recognition became less theoretical, more vivid, and integrated into everyday life after performing the life story interview. Hanne described it like this:

I have in a way been very conscious about that there is a person behind the diagnosis…but what changed with the life story interview was that I became much more deliberate of it…I could somehow picture it.

The participants expressed that the life story interview provided the basis for a different engagement than the “life story template” they use in the nursing documentation system. They described the life story interview as more in-depth and personal, although it was also perceived as less practical. The life story interview was something the participants only conducted once, yet they added a “life story perspective” into their everyday work–life. The extent of the impact of doing the interview was expressed by Lene: “*It, sort of, sneaks into the way you think and the way you talk with patients.”*

The “life story perspective” that the participants describe summarizes their renewed interest, experience of fellowship, engagement, and understanding of older peoples’ lives both in the past and the present.

## Discussion

### Engaging Fellowship

An important finding in the study was that life story work can foster a renewed interest in getting to know the individual person, expressed through the participants asking more than they did before. The desire to share one's story is based on the belief that what you are telling is interesting to the person listening (Hansen, 2016). Martinsen's *sensuousness* also requires genuine interest (Martinsen, 2000). As an extension of this interest, the study shows that the participants listen more than they did before. Although this is specifically said about listening to the patients’ stories, it is transferable to other conversations and settings. Someone who is present and listens attentively, will—in our opinion—also be more receptive to other aspects of *sensuousness*. When you are already listening, it is easier to also see, smell, feel, and listen to signals that are not explicitly uttered. *Sensuousness* makes it more difficult to distance oneself from the patient, “capturing” us with its human authenticity (Austgard, 2010).

Furthermore, the participants in the study experienced that it is possible to create a deeper connection with the patients despite having limited time available. Being met as a fellow people is not necessarily about the amount of time available for the meeting, but it is rather about being open to receiving the other and meeting the other with an attentive, responsive eye (Martinsen, 2000). This is supported by Kvåle's (2006) study, in which patients expressed that care had more to do with presence than with time. Moreover, Martinsen (2000) points out that professional judgment—which requires attentive presence—performs poorly when time pressure prevails.

The study also shows that conversations with patients can be characterized by “autopilot.” In that case, the nurse's questions emerge from what he or she thinks in advance they *should* ask about based on their own knowledge, experience, and preconceptions. Martinsen’s description of *holding back* will sometimes be about this: to withhold one's own prior knowledge and experiences to let something else, such as the patient's perspective and person, emerge. Martinsen also emphasizes that there is no contradiction between being a professional and being personally involved. Being a professional is about daring to let the relevant other emerging as an equal (Martinsen, 2000).

Through the life story interview, the nurses in this study experienced conversations with the patients that were less nursing- and disease-related. They describe more focus on the patient's personal life and what is important to the individual. They also describe being less “stuck” in the role of a nurse. The striving to be a professional nurse—one who masters the situation—can make carrying out what Martinsen writes about daring to be quiet and stay present in uncertainty be difficult. The desire for professionalism is legitimate, but if it triggers a need for control by “mastering” the patient, then one is not acting in the patient´s best interest (Martinsen, 2003). The participants in this study express that the patients, through their life stories, appear to them more clearly as a *person*, thus becoming more than a diagnosis, task, or patient. Other studies on the use of life story in older people care also show that hearing a life story helps health workers to see the person behind the patient (Cooney & O'Shea, 2019; Grøndahl et al., 2017; McKeown et al., 2010). Mentally placing the patient as a diagnosis or task can be a tool to gain control of the situation and “master” the other. The results of this study show that life story work can help counteract such reductionism and foster connectedness with matters closer to the soul.

An important finding in the study shows that sharing a life story can promote the experience of fellowship between nurse and patient. Other studies on the use of life story also point to a more relationship-focused version of care (Cooney & O'Shea, 2019; Gridley et al., 2016; Grøndahl et al., 2017). In Martinsen's thinking, our lives do not become meaningful in isolation, it is through our engagement with others that life gains its significance (Austgard, 2010). Fellowship lies in the very nature of caregiving. The experience of fellowship creates an “us” and reduces the distance between people, making the work of a nurse meaningful.

The fact that the patients are old and frail/helpless is a factor that creates distance between them and others. This can be interpreted in the context of a general emergence of ageism in society (Daatland, 2008). Martinsen (2011) writes about the cultural attitude in which we do not recognize frailty, dependence, and death as natural parts of life and being human. It is almost shameful to be a frail patient who is dependent on others. To meet people in their frailty, nurses must be able to recognize their own vulnerability (Martinsen, 2012). Otherwise, if nurses are afraid of becoming frail themselves that will create an unnatural distance from the patient and prevent fellowship and connection. The results of the study show that the participants have become more aware of the stigma associated with being old and how that stigma affects them and their work.

Other studies also indicate that awareness-raising has a positive effect on age prejudice (Burnes et al., 2019; Even-Zohar & Werner, 2020; Lunde, 2017). Furthermore, it appears from the results that hearing an older person's life story can emphasize the understanding and experience that older people are just as unique as anyone else. Their unique humanity emerges more clearly through life story (Cooney & O'Shea, 2019). The results also show that life story has helped the participants see that being old and frail certainly is not synonymous with being uninteresting.

The study reveals that sharing a life story promotes recognition and empathy for the patient's situation. This finding is also confirmed by other studies that have used life story (Gridley et al., 2016). For Martinsen (2012), the ability to become involved is a natural part of care. Involvement requires the ability to see the other as my fellow human being and the ability to be *warmly engaged*. The participants point out that when listening to the life stories, they could recognize themselves because the stories were emotionally engaging. Recognition confirms that the story is real, based on real human experience. Martinsen (2000) also points out that involvement and warm engagement require that we be open to being emotionally touched. The participants experienced being affected through life story work and now when they meet patients, they are more open to emotional touch.

It is our understanding, based on the presented results, that life story work awakens and strengthens a nurse's ability to provide care with *sensuousness* and *warm engagement* and the ability to *hold back*. Nurses’ interest and recognition of the individual patient reinforce their ability to build close relationships that create engaging fellowship between the nurse and the patient. Everything relates to what Martinsen describes as the mastery of “seeing with the heart’s eye” (2006, p. 83).

### Understanding the Importance of Life Story Work

The results show that life story work can provide a deeper understanding of the older generation and more focus on the whole person. Maintaining the patient's integrity, which means being whole, is a particular challenge in meeting the needs of older frail patients (Kirkevold et al., 2020). Furthermore, we find that being met with respect and dignified care that acknowledges who one is as an individual person is very important for older people receiving municipal healthcare in everyday life—both at home and in institutions (Rykkje, 2018). A whole person approach is about the awareness that the person we meet is more than what immediately meets the eye (Austgard, 2010). Still, it is important to point out that in human complexity there will always be much that is hidden from us (Alvsvåg, 1997). In this sense, holistic nursing can never be fully holistic because a part of the whole will always be beyond the horizon of our vision and understanding.

The participants describe gaining a greater understanding of “the little things.” They emphasize that the little things can be very important to the person concerned. Providing the little things that matter to patients requires time, sensitivity, empathy, and concern (Williams et al., 2016). The respect for what is valuable to the patient has a sound base in Martinsen's view of *holding back*, where the nurse allows the patient's perspective to be at the center of a given situation (Martinsen, 2010). The little things can also trigger big emotions and represent deeper values that are points of reference in the building of relation between nurse and patient.

When the ideas of *sensuousness* and *holding back* are combined with the life story, which is possible in the context of the life story interview and “life story perspective,” then much of the criticism of life story work (McKeown et al., 2015) no longer seems applicable. The older persons, interviewed by nurses in this study, were telling their own stories and were able to choose freely what to include and exclude. Nevertheless, the “life history perspective” in everyday life is not without ethical challenges. A need for caution must be mentioned, as many people do not want to share their story. Again, the necessity of Martinsen's *sensuousness* and *holding back* is made clear. It is important to assess the life story’s place in the given situation and let the conversation and the story emerge on the patient's own terms.

The study suggests that the nurses’ changes in interest, experience of fellowship, engagement, and understanding do not only apply to the person the participants interviewed in the life story interview. The renewed insight and understanding are transferred to patients more generally. The life story interview itself was conducted once, yet it gave the nurses a “life story perspective” in their daily work, which meant focusing on the people they meet in their workday as fellow human beings. Such a perspective in the work of a nurse is professionally advantageous, as a life story gives us unique insight into what is important and valuable for the narrator (Fossland & Thorsen, 2010; Medeiros, 2014). The perspective is also beneficial from a human point of view, which one of the participants described as being that she experiences herself becoming more human by coming to see her patients as people rather than merely as patients, diagnoses, and work tasks. Studies of patients’ experiences and what they choose to emphasize when meeting with healthcare professionals show that, first and foremost, the patients wanted to be treated as fellow human beings (Atkins, 2006; Eldh et al., 2006; Johannessen & Steihaug, 2019; Kvåle, 2006; Nakrem et al., 2013; Struhkamp, 2005).

Listening to a person's life story is a powerful way to show that they are valued as human beings. Several studies on the use of life story work in older people's care indicate that learning a life story helps to see the person behind the patient (Bakken et al., 2009; Cooney & O'Shea, 2019; Grondahl et al., 2017; McKeown et al., 2010). The participants also had an awareness of this before the life story interview. Nevertheless, they describe how easy it is to slip into a more mechanical way of working, and they suggest that the life story interview became a “wake-up call” that drew the “fellow people perspective” closer to the front of their consciousness. This is, in our opinion, a very important point. What is at the forefront of the nurse's consciousness in the meeting with patients will affect the nurse's actions, choices, and priorities (Martinsen, 2018). If the “completion of the work task” is in focus, there will not necessarily be room for a view of the other person as a person in that perspective. On the other hand, if the person and their needs are in focus, then the nurse will be present with *sensuousness*, expressing being open to and available for the other. Openness to the narratives in everyday life can lead nurses to encourage older patients to express their experiences (Blix et al., 2021); thus, this will add a deeper connection and warmth to the relationship between caregivers and patients. We believe the nurses become more open to accepting the patient as a fellow human being, which again safeguards the patients’ sense of dignity in old age (Rykkje, 2018).

Gadamer (2004) describes the essence of understanding as being that something happens to us when we experience something to be true: an opinion arises. The understanding the participants have gained is not primarily intellectual. Theoretically, the participants knew in advance that the patients had lived long lives and experienced a lot. Their new understanding is based on the experience of conducting the life story interview as having made the patients’ pasts feel more real and relevant. The experience settled in the participants' bodies, and the “life story perspective” was moved closer to the front of their consciousness in their daily work.

### Strengths and Limitations

The number of participants in the study is small; however, the data material is rich and sufficient to gain valuable insight according to philosophical hermeneutics (Gadamer, 2004). The participants were students in further education, which made it difficult to distinguish between which experiences and considerations were a consequence of the current life story interview and those resulting from the general development and educational formation process. On the other hand, the formation process made the participants more aware and able to reflect on their own practice, which added insights and rich descriptions in the interviews.

The participants were recruited from a further education program in gerontology where all students were females. This reflects the gender imbalance in older people care, thus we consider that the findings are transferable to practice. However, we acknowledge that it would be valuable to hear the voices of health professionals with different cultural backgrounds and all genders.

The researchers have the same profession and field as the participants and have previously conducted life story interviews with older people. Strong identification with the participants can be a pitfall, as it can create a challenge in maintaining sufficient professional distance (Kvale & Brinkmann, 2017). The researchers were aware of this as part of the preunderstanding and thus, the hermeneutical reading of the stories was focused on letting the text's meaning emerge as freely as possible. Awareness of our preconceptions as researchers has been central throughout the different stages of the research process. From a hermeneutic perspective, every researcher will bring with them a unique perspective and preconceptions. A study “from within,” as in our study, can also provide important insights and new knowledge. As Gadamer (2004) points out, our prejudices (previous knowledge and experience) do not have to be an obstacle to understand the perspective of the participant. It is our understanding that the researchers’ prejudices can support new understanding through a hermeneutical dialogue with the interview text. That means that we tried to understand the individual viewpoint of the participants; however, we also acknowledge that the findings are based on our preunderstanding of life story work and its significance in older people care.

## Implications for Practice

*Sensuousness* and hermeneutics can be related; we use our senses and interpret our impressions on the basis of our own preunderstanding. The change in focus that the participants describe can also be seen in the light of Gadamer's horizon of understanding, which is constantly changing and expanding through the inclusion of new experiences and the integration of those experiences (Gadamer, 2004). The patient you meet is a fellow human: a real human being, who laughs, cries, and loves. This perspective seems to become more alive for the participants through life story work.

## Conclusion

The power of the narrative is revealed in this way because it resonates with real and recognizable human experience. It makes it almost impossible to distance oneself from being engaged with the patient's life. Knowledge of human life and history makes it easier to experience their version of life (Lee et al., 2020). Alvsvåg (1997) writes that our view of humanity, consciously or unconsciously, settles in the body and will always appear in our encounters with others. Our view of humanity, at depth, also emerges in the nurse's care for their patients. Fundamentally, seeing the patient as an irreplaceably unique fellow human being and having the person's dignity close to our consciousness in daily care is, in our opinion, essential in nursing care. The consequences of the presence and absence of this consciousness echoes through the words of Martinsen (2012, p. 12):

(…) we [can] only with our attitude to another person make that person's world feel insecure, we can make the freshness wither. But we can also help to make the other's world broad, bright, diverse and safe.
